# Novel Localization of Peripherin 2, the Photoreceptor-Specific Retinal Degeneration Slow Protein, in Retinal Pigment Epithelium

**DOI:** 10.3390/ijms16022678

**Published:** 2015-01-26

**Authors:** Patrizia B. Uhl, Barbara Amann, Stefanie M. Hauck, Cornelia A. Deeg

**Affiliations:** 1 Institute for Animal Physiology, Department of Veterinary Sciences, Ludwig-Maximilians-University, Veterinärstraße 13, D-80539 Munich, Germany; E-Mails: p.uhl@tiph.vetmed.uni-muenchen.de (P.B.U.); baerbl.amann@tiph.vetmed.uni-muenchen.de (B.A.); 2 Research Unit for Protein Science, Helmholtz Zentrum München, German Research Center for Environmental Health GmbH, Ingolstädter Landstr. 1, D-85764 Neuherberg, Germany; E-Mail: hauck@helmholtz-muenchen.de

**Keywords:** peripherin 2, retinal pigment epithelium, retina, rhodopsin, uveitis

## Abstract

Retinal pigment epithelium (RPE) builds the outer blood-retinal barrier of the eye. Since one typical feature of the autoimmune disease, equine recurrent uveitis (ERU), is the breakdown of this barrier, we recently performed comparative analysis of healthy and uveitic RPE. We identified for the first time peripherin 2, which is responsible for visual perception and retina development, to be localized in RPE. The purpose of this study was therefore to validate our findings by characterizing the expression patterns of peripherin 2 in RPE and retina. We also investigated whether peripherin 2 expression changes in ERU and if it is expressed by the RPE itself. Via immunohistochemistry, significant downregulation of peripherin 2 in uveitic RPE compared to the control was detectable, but there was no difference in healthy and uveitic retina. A further interesting finding was the clear distinction between peripherin 2 and the phagocytosis marker, rhodopsin, in healthy RPE. In conclusion, changes in the expression pattern of peripherin 2 selectively affect RPE, but not retina, in ERU. Moreover, peripherin 2 is clearly detectable in healthy RPE due to both phagocytosis and the expression by the RPE cells themselves. Our novel findings are very promising for better understanding the molecular mechanisms taking place on RPE in uveitis.

## 1. Introduction

Autoimmune uveitis is a sight-threatening disease mediated by autoreactive T-cells that cross the blood-retinal barrier with subsequent inflammation of the inner eye and destruction of retinal structures [1]. Until now, molecular mechanisms of pathophysiology leading to the loss of the immune-privilege of the inner eye are not fully understood. The only spontaneous animal model for autoimmune uveitis in man is equine recurrent uveitis (ERU), a remitting-relapsing disease of horses that results in blindness [2,3]. There are many similarities in clinical presentation, the course of the disease and autoimmune reactions between human autoimmune uveitis patients and ERU cases [2,4]. These resemblances and the fact that intraocular tissue samples are available from horses in all stages of disease, which have an outbred genetic background and are exposed to all typical environmental conditions, render ERU an ideal model for uveitis in man. Thus, investigating pathomechanisms contributing to this frequently occurring eye disease of horses has become very important [2,5].

Retinal pigment epithelium (RPE) builds the outer blood-retinal barrier [6] and is the crucial part of the immune defense of the eye [7]. We started with in-depth molecular analysis of RPE cells by creating an equine cell line and comparing the cell surface proteome of Passage 4 equine RPE cells and native cells [6]. Since there was a significant alteration between cell surface proteomes of native and cultured cells, the suitability of cultured cells for molecular analyses was affected [6]. Thus, in a follow-up study, we used *in situ* labelling of tissue samples in very short post-mortem times, which enabled the identification of 148 RPE cell surface proteins, potentially representing their appearance in physiological and pathophysiological *in vivo* processes [5].

Among these 148 proteins, 27 were differentially expressed between healthy and ERU cases [5]. Surprisingly, one of the differentially expressed proteins, detected in equine RPE, was peripherin 2, which is, under normal physiological conditions, responsible for visual perception and retina development [5]. Peripherin 2 is known to be selectively expressed in the photoreceptor outer segment of the retina [8], and mutations in the *peripherin 2* gene are commonly associated with retinal degenerative diseases, like retinitis pigmentosa and autosomal dominant macular dystrophy in man [9]. However, until now, there was no information about peripherin 2 expression in RPE and whether expression changes of peripherin 2 were associated with autoimmune uveitis.

Therefore, the goal of this study was to validate our novel finding of peripherin 2 localization to equine, as well as to human RPE cells and to characterize the respective expression patterns of peripherin 2 in physiological and uveitic equine eyes.

## 2. Results

### 2.1. Peripherin 2 Locates to the Photoreceptor Outer Segments of Equine Retina with no Significant Difference between Healthy and ERU Diseased Samples

**Figure 1:**
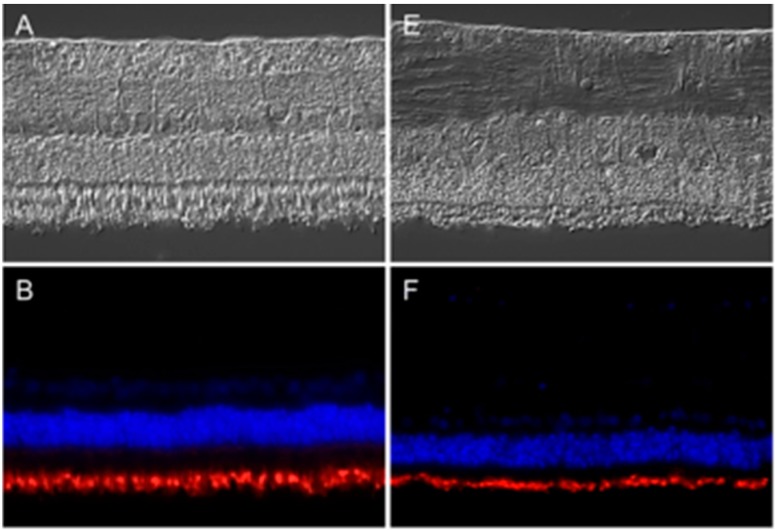

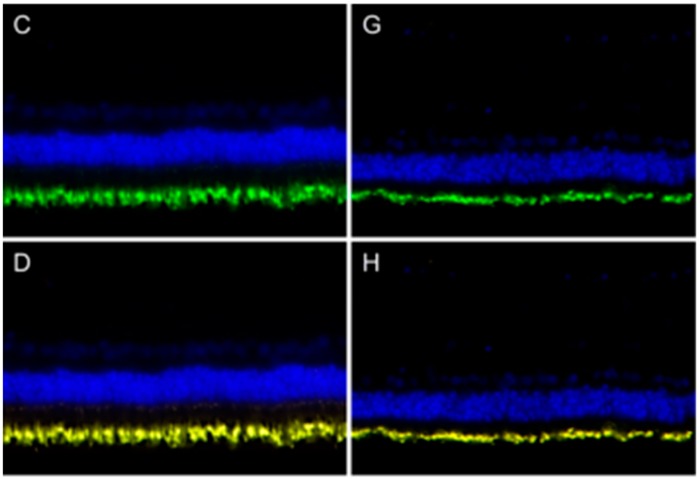
Expression pattern of peripherin 2 (red) and rhodopsin (green) in representative retinas of healthy (**left**) and equine recurrent uveitis (ERU) cases (**right**). Differential interference contrast image of healthy (**A**) and diseased (**E**) equine retina. Peripherin 2 (red) was equally distributed in the photoreceptor outer segments of healthy (**B**) and diseased (**F**) equine retina. Cell nuclei were stained with 4',6-diamidino-2-phenylindol (DAPI) (blue); Rhodopsin (green) was also equally distributed in photoreceptor outer segments of healthy (**C**) and uveitic (**G**) retinas. The overlap of peripherin 2 and rhodopsin in healthy (**D**) and uveitic (**H**) retina show overlapping expression pattern of both proteins (yellow).

Mutations in the *peripherin 2* gene are associated with many human retinal degenerative diseases, like retinitis pigmentosa and adult vitelliform macular dystrophy [8]. Therefore, and due to the fact that, to our knowledge, there was no information about the expression pattern of peripherin 2 in equine retina, we were interested in assessing its physiological expression pattern, as well as its disease-associated changes. We thus performed antibody-based staining of healthy and uveitic retinal sections (Figure 1). Differential interference contrast (DIC) images show representative retinas of healthy (Figure 1A–D) and diseased (Figure 1E–H) horses. In healthy equine retina, peripherin 2 was detectable in photoreceptor outer segments with equal intensity along the whole intact photoreceptor layer (Figure 1B, peripherin 2 = red). Interestingly, the same expression pattern was found in ERU diseased retinas (early stages), where peripherin 2 was also localized in the thinner, but still completely intact, photoreceptor outer segments with a similar intensity and distribution (Figure 1F, peripherin 2 = red) as in healthy equine retinas (Figure 1B, peripherin 2 = red). Since rhodopsin is a typical photoreceptor marker [10], we were interested in its expression pattern in healthy and uveitic equine retinae compared to the expression pattern of peripherin 2. Therefore, sections stained with anti-peripherin 2 were also stained with anti-rhodopsin. Like peripherin 2, rhodopsin was detectable in photoreceptor outer segments with an equal distribution and intensity in healthy retina sections (Figure 1C, rhodopsin = green). The same expression pattern of rhodopsin was found in ERU diseased retinas (Figure 1G, rhodopsin = green). In addition, the overlay of peripherin 2 and rhodopsin showed the same expression pattern in healthy (Figure 1D, peripherin 2 = red, rhodopsin = green, overlap of peripherin 2 and rhodopsin = yellow), as well as in uveitic retinae (Figure 1H, peripherin 2 = red, rhodopsin = green, overlap of peripherin 2 and rhodopsin = yellow).

### 2.2. Peripherin 2 Is Detectable in Healthy Equine RPE Cells and Downregulated in Uveitic Cases

**Figure 2:**
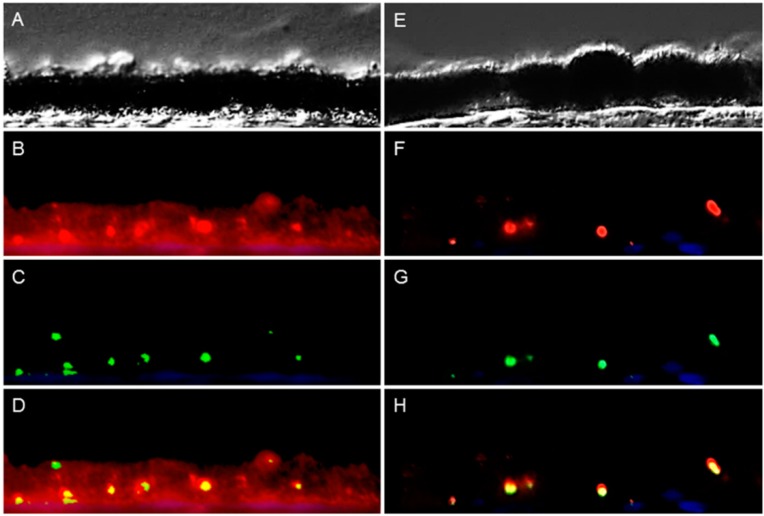
Peripherin 2 (red) and rhodopsin (green) expression in retinal pigment epithelium (RPE) of representative healthy (**left**) and ERU cases (**right**). Differential interference contrast image of healthy (**A**) and diseased (**E**) equine RPE. In healthy (**B**) RPE, peripherin 2 was expressed over the entire RPE cell layer, and the intensity was enhanced in some spots. In uveitic RPE cells (**F**), peripherin 2 was significantly reduced to few small spots; Rhodopsin was limited to few isolated spots, distributed throughout the whole RPE layer, in healthy (**C**), as well as in diseased (**G**) RPE. The overlap of peripherin 2 and rhodopsin in healthy (**D**) RPE indicates significant higher expression of peripherin 2 compared to rhodopsin (the yellow overlap is only displayed in a few spots). In uveitic RPE (**H**), peripherin 2 was only expressed at rhodopsin expression sites (yellow spots).

Peripherin 2 was known to be localized in photoreceptor outer segments of mouse, rat and bovine retina [11,12]. In this study, we found a similar expression pattern in photoreceptor outer segments in horse retina (Figure 1). Interestingly, in a prior study, we detected peripherin 2 on the cell surface of RPE cells using *in situ* biotinylation followed by LC-MS/MS [5]. Since the peripherin 2 expression pattern in RPE was not described yet, we performed immunohistochemistry to localize peripherin 2 in healthy equine RPE and to examine ERU-associated changes in diseased equine RPE (Figure 2). In order to exclude the correlation of decreased peripherin 2 expression with the destruction of diseased RPE cells in advanced stages of ERU, we only investigated morphologically intact RPE of ERU cases. Thus, DIC images of healthy (Figure 2A) and diseased (Figure 2E) equine RPE showed a morphologically intact RPE monolayer in both cases. In healthy equine RPE, peripherin 2 was expressed across the whole cell and along the whole RPE layer, excluding the apical rim (Figure 2B, peripherin 2 = red). In addition, the intensity of peripherin 2 was constant throughout the entire healthy RPE with some enhanced spots (Figure 2B, peripherin 2 = red). Interestingly, immunohistochemical staining of RPE of diseased horses revealed a significant reduction of peripherin 2 expression in uveitic RPE (Figure 2F, peripherin 2 = red, the same specimen as shown in Figure 1E–H). Accordingly, expression changes of peripherin 2 selectively concerned RPE cells, but not photoreceptor outer segments in ERU cases (Figure 1E–H and Figure 2E,F, the same ERU case). Peripherin 2 expression was limited to a few small spots in diseased RPE, and the intensity of peripherin 2 was significantly reduced (Figure 2F, peripherin 2 = red) compared to healthy controls (Figure 2B, peripherin 2 = red). Since RPE cells are the most active phagocytic cells in the body [10], it seemed obvious that peripherin 2 gets into RPE by phagocytosis. In order to pursue this thought, we double stained RPE sections for peripherin 2 and rhodopsin (Figure 2). We decided to use rhodopsin, because it is known to be phagocytized within photoreceptor outer fragments by RPE, making rhodopsin a suitable marker for phagocytic processes [13,14]. Both in healthy (Figure 2C, rhodopsin = green) and in uveitic (Figure 2G, rhodopsin = green) RPE, rhodopsin was typically concentrated in a few spots, which were distributed over the whole RPE layer. In contrast to peripherin 2 (Figure 2B,F, peripherin = red), the rhodopsin localization pattern was unchanged in uveitic RPE compared to healthy controls. Regarding the overlap of the two proteins, it becomes apparent that the spots expressing rhodopsin were also clearly positive for peripherin 2 (Figure 2D,H, overlap of peripherin 2 and rhodopsin = yellow). Besides, peripherin 2 was constantly expressed throughout the entire remaining parts of healthy RPE (Figure 2D, peripherin 1 = red, rhodopsin = green, overlap of peripherin 2 and rhodopsin = yellow). In contrast, in uveitic RPE, downregulation of peripherin 2 was clearly detectable, and only spots stained positive for rhodopsin were also positive for peripherin 2 (Figure 2H, overlap of peripherin 2 and rhodopsin = yellow).

### 2.3. Expression Changes of Peripherin 2 Selectively Concerned RPE Cells, but not Photoreceptor Outer Segments in ERU Cases

**Figure 3:**
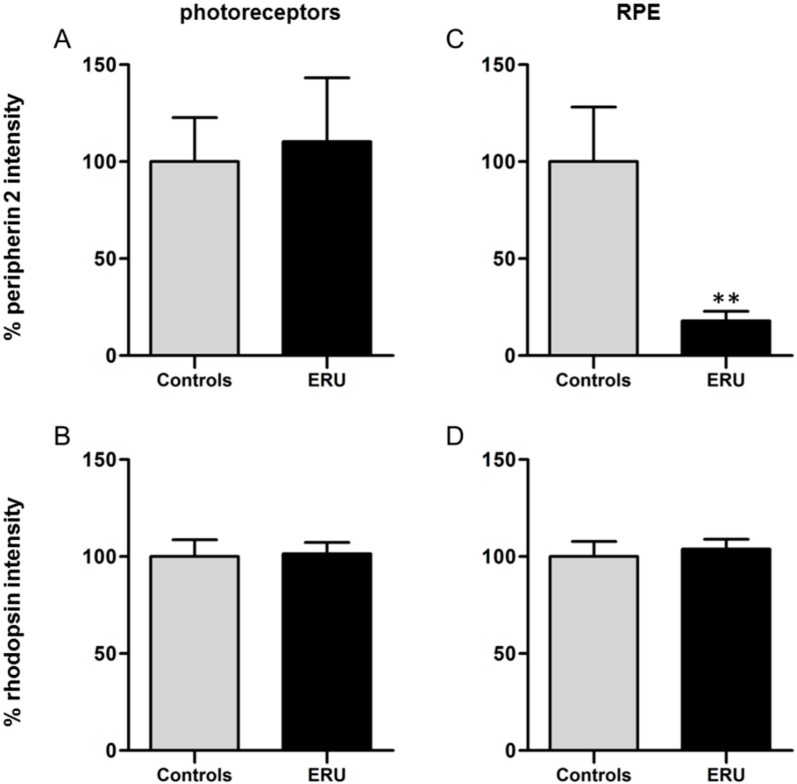
(**A**) Quantification of the fluorescence intensity of peripherin 2 expression in photoreceptor outer segments revealed no significant difference between healthy (grey column) and ERU cases (black column); (**B**) There was also no significant difference between the fluorescence intensity of rhodopsin in photoreceptor outer segments of healthy (grey column) and uveitic (black column) retinas detectable; (**C**) A significant reduction of the fluorescence intensity of peripherin 2 in uveitic RPE to 17.9% (black column), compared to RPE of negative controls (** *p* ≤ 0.01); (**D**) Quantification of the fluorescence intensity of rhodopsin in RPE showed no significant difference between healthy (grey column) and ERU cases (black column).

To quantify the expression of peripherin 2 and rhodopsin, fluorescence intensities of healthy and uveitic retinas and RPE were measured and analyzed (Figure 3). A comparison of the fluorescence intensity of peripherin 2 in healthy retinas (Figure 3A, grey column, 100% ± 22.8%) and uveitic retinas (Figure 3A, black column, 110.4% ± 32.9%) revealed no significant difference of protein expression in photoreceptors. A similar result was shown for the quantification of the fluorescence intensity of rhodopsin in healthy (Figure 3B, grey column, 100% ± 8.6%) and uveitic (Figure 3B, black column, 104.1% ± 5.8%) photoreceptor outer segments. In addition, rhodopsin expression in healthy RPE (Figure 3D, grey column, 100% ± 7.8%) did not significantly differ from uveitic RPE (Figure 3D, black column, 103.9% ± 5.1%). In contrast, the mean fluorescence intensity of peripherin 2 in equine RPE cells of ERU cases (Figure 3C, black column) was significantly reduced to 17.9% ± 5% (** *p* ≤ 0.01) of the physiological expression in healthy controls (Figure 3C, grey column, 100% ± 28.1%).

### 2.4. Cultivated RPE Cells still Express Peripherin 2

**Figure 4:**
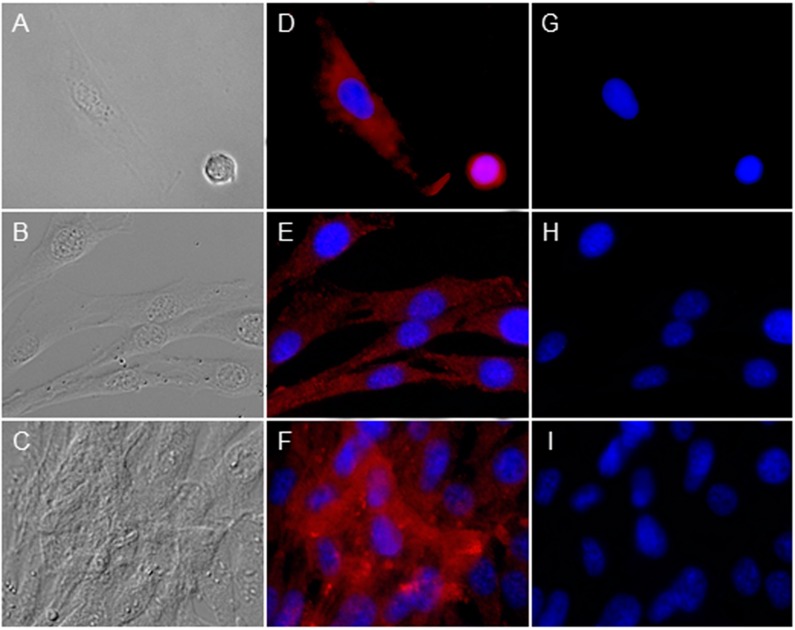
Investigation of peripherin 2 (red) and rhodopsin (green) expression on cultivated healthy RPE cells at different confluency states. Differential interference contrast image of healthy equine RPE cells at different confluency states; from single cells (**A**) to 40% confluent cells (**B**) up to 90%–100% confluent cells (**C**); (**D**–**F**) Peripherin 2 (red) was expressed over whole RPE cells with an even intensity (cell nuclei = blue); (**G**–**I**) No rhodopsin (green) expression was found in primary healthy equine RPE cells (cell nuclei = blue).

To investigate whether RPE cells express peripherin 2 by themselves or if the protein got into RPE cells just by phagocytosis, we investigated the peripherin 2 expression pattern of cultivated, primary healthy RPE cells (Figure 4). Differential interference contrast image shows healthy RPE cells (Figure 4A–C) at different confluency states, from single cells (Figure 4A) up to confluent cells (Figure 4C). In each growth stage, peripherin 2 expression was clearly detectable over the whole RPE cell with an equal intensity (Figure 4D–F, peripherin 2 = red). Furthermore, we stained these healthy RPE cells also for rhodopsin to examine the appearance of rhodopsin compared to peripherin 2 in cultivated RPE cells. In contrast to peripherin 2, rhodopsin was not detectable in passaged RPE cells (Figure 4G–I).

### 2.5. Peripherin 2 Is also Expressed by Human RPE

**Figure 5:**
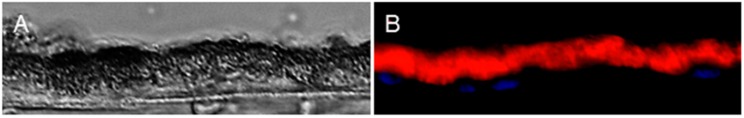
Peripherin 2 (red) expression in healthy human RPE. (**A**) Differential interference contrast image of healthy human RPE; (**B**) peripherin 2 (red) was expressed over the whole RPE layer with an even intensity.

To strengthen our novel finding of peripherin 2 existence in RPE, we wanted to investigate whether the expression of peripherin 2 was also detectable in human RPE cells. Therefore, we detected peripherin 2 expression with immunohistochemistry in human RPE sections. Peripherin 2 staining of human RPE sections corresponded very well with our latest findings of the peripherin 2 distribution in equine RPE. As in healthy equine RPE, peripherin 2 was clearly detectable over the whole RPE layer with an equal intensity in human RPE (Figure 5, peripherin 2 = red).

## 3. Discussion

Pathogenesis and etiology of ERU, the only spontaneous animal model for human autoimmune uveitis, are not entirely clarified to date [3,15]. However, differential proteome analyses provided more insights into the molecular pathomechanisms of this spontaneously occurring disease by detecting several differentially-expressed proteins in uveitic vitreous, retina, sera and infiltrating leukocytes, which might be crucially involved in disease development and progression [2,16,17,18,19,20]. Since one characteristic feature of ERU is the breakdown of the outer blood-retinal barrier [21], the RPE is significantly involved in the pathophysiological processes of uveitis. Thus, we recently performed comparative analysis of the plasma cell membrane proteomes of healthy and uveitic RPE cells *in situ* to gain a better understanding of the pathomechanisms leading to the breakdown of the blood-retinal barrier [5]. Interestingly, one of the differentially-expressed plasma cell membrane proteins was peripherin 2, which is, under normal physiological conditions, responsible for visual perception and retina development (according to GO terminology). Peripherin 2 was found to be substantially downregulated in ERU cases [5]. In the present study, we were now able to validate this novel localization of peripherin 2 in physiological equine RPE (Figure 2) and its significant downregulation in ERU (Figure 2 and Figure 3). Moreover, we were able to detect peripherin 2 in human RPE (Figure 5). Then, we further characterized the expression pattern of peripherin 2 in equine RPE, and thereby, we could show the localization of peripherin 2 in the whole healthy RPE cell, whereas in our previous study, we only knew about the occurrence in the plasma cell membrane [5]. Furthermore, we demonstrated that peripherin 2 is localized with an even intensity in the photoreceptor outer segments of rods and cones in physiological equine retina (Figure 1). So far, a similar expression pattern of peripherin 2 within photoreceptor outer segments was detected in other species, like cows, rats and mice [12,22].

Even more interesting, in our point of view, is the novel finding of peripherin 2 expression in equine (Figure 2) and human (Figure 5) RPE cells. To our knowledge, the localization of peripherin 2 in RPE was not described in other species so far. One question was how peripherin 2 gets in this position. Since one of the main functions of RPE cells is phagocytosis of photoreceptor outer fragments (10% per day) [13], it seemed obvious that peripherin 2 is phagocytized and, hence, to be found in the phagosomes of RPE cells. As rhodopsin is a major protein in photoreceptor outer segments, it is already known to be phagocytized by RPE cells and, therefore, used as a marker for phagocytosis activity [14]. Thus, we investigated possible co-localization of rhodopsin and peripherin 2 in equine RPE, which would indicate an association of peripherin 2 with RPE phagocytosis. Double-stainings of rhodopsin and peripherin 2 in healthy RPE revealed peripherin 2 distribution over the whole RPE layer, whereas rhodopsin was concentrated to a few spots (Figure 2). This localization pattern of peripherin 2 and rhodopsin led to the following assumption: spots staining positive for rhodopsin are presumably phagosomes, containing photoreceptor outer segment waste, including rhodopsin. As peripherin 2 was also localized in these spots, it can be concluded that peripherin 2, like rhodopsin, is phagocytized by RPE cells. Recently, the co-localization and interaction of peripherin 2 and rhodopsin in photoreceptor outer segments were detected for the first time by *in vitro* (HEK293 cells) and *in vivo* (mouse retina) co-immunoprecipitation and fluorescence resonance energy transfer [23]. It was shown that peripherin 2 was required as a molecular linker between rhodopsin and the rod cyclic nucleotide-gated channel [23]. Thereby, peripherin 2 binds to rhodopsin with high affinity, comparable to that of rhodopsin dimers [23]. These findings further strengthen our thesis that peripherin 2 is phagocytized in the same manner as rhodopsin by RPE cells.

However, we found peripherin 2 not only in co-localization with rhodopsin, but also equally distributed over the whole healthy RPE (Figure 2). Therefore, it seems obvious that peripherin 2 is additionally expressed by RPE cells. To prove this presumption, we stained healthy cultivated RPE cells with anti-peripherin 2 and anti-rhodopsin antibodies. In contrast to rhodopsin, which was not found in cultivated RPE cells, peripherin 2 was detectable over the whole cell (Figure 4). In conclusion, peripherin 2 gets into RPE cells by phagocytosis, but is additionally expressed by healthy RPE cells themselves.

In uveitic RPE sections, no difference between rhodopsin and peripherin 2 localization was detectable, and overlapping of both stainings was found in a few spots, mainly localized in the basal side of RPE layer (Figure 2). Thus, localization of phagosomes in healthy compared to uveitic RPE leads to another interesting finding: there was no detectable alteration in the RPE engulfment capability of ERU diseased horses in the stages of disease, in which RPE cells are morphologically still intact. However, whether the degradation of photoreceptor outer segments is affected in the case of uveitis or not has to be clarified in further studies by *in vitro* phagocytosis assays, for example. A model for an eye disease in which altered phagocytosis activity of diseased RPE cells was associated with pathomechanisms leading to progressive and irreversible central vision loss is Best’s disease, an inherited degenerative disease of human macula, which is caused by mutations in the RPE gene, bestrophin 1 [24]. By performing phagocytosis assays with rhodopsin, a delayed degradation of photoreceptor outer segments occurred in RPE cells of Best disease compared to controls, which led to reduced clearance of photoreceptor outer segments [24].

Also of high interest are the known functional features of peripherin 2 in photoreceptors, like the responsibility for tissue morphogenesis and stability, as well as membrane fusion processes [23], since they are also very important functions of RPE. Therefore, downregulated peripherin 2 in uveitic RPE (Figure 2 and Figure 3) could lead to functional restrictions of RPE in ERU cases. To what extent peripherin 2 is associated with the breakdown of the blood-retinal barrier in uveitic cases is still being investigated. Interestingly, even though peripherin 2 was never described with changed expression in uveitis before, it is very often involved in retinal pathologies, such as adult-onset foveomacular vitelliform dystrophy, pattern dystrophies, cone-rod dystrophies, retinitis pigmentosa and retinitis punctata albescens through mutations of the *peripherin 2* gene [11,25]. The normal product of the *peripherin 2* gene, the protein peripherin 2, was for the first time identified in 1992 by cDNA sequence analysis [26]. Up to now, more than 150 mutations in the *peripherin 2* gene, leading to these retinal diseases, have been identified (available online: http://www.retina-international.org/files/sci-news/rdsmut.html). Depending on the particular mutation, varying retinal degenerations develop. Most closely to our findings that peripherin 2 expression is downregulated in uveitis (Figure 2 and Figure 3), the nmf193 mutant mouse model, exhibiting a single base change in the *peripherin 2* gene, showed the downregulation of peripherin 2 in photoreceptor outer segments [27]. In this model, downregulation of peripherin 2 expression may directly contribute to the pathology of photoreceptor degeneration [27]. These nmf193 mutant mice show photoreceptor outer segment defects and progressive retinal degeneration [27]. Since the same reduction of peripherin 2 and similar photoreceptor degeneration of the nmf193 mutant was also found in another, non-peripherin 2 gene-associated mouse mutant of the photoreceptor-specific nuclear receptor gene, Nr2e3, a direct connection between decreased peripherin 2 expression and photoreceptor degeneration was concluded [27]. This direct association between decreased [27] or absent peripherin 2 expression [28] and the degeneration of photoreceptor cells suggests that also the reduction of peripherin 2 in diseased RPE cells could lead to the damage of RPE cells followed by disruptions of cell-cell junctions with consequently increased permeability of the outer blood-retinal barrier. To what extent peripherin 2 reduction in uveitic RPE is essentially associated with the mechanisms leading to blood-retinal barrier breakdown in the case of ERU is yet to be clarified. Therefore, one next step could be to perform leukocyte transmigration assays through a RPE monolayer knocked down for peripherin 2 using siRNA.

## 4. Experimental Section 

### 4.1. Retina and RPE Specimen

For this study, the eyes of 19 horses (11 healthy controls and 8 spontaneous ERU cases) and of one human donor without a known history of eye disease were used. In detail, the eyes of 15 horses (7 healthy controls and 8 spontaneous ERU cases) and of one human donor were used for immunohistochemical stainings of retina and RPE sections, and 4 healthy equine eyes were used for immunocytochemical stainings. The specimens of healthy equine eyes and ERU cases were obtained from horses that had to be euthanized due to causes unrelated to this study. The collection and use of equine eyes from animals that were killed due to a research-unrelated cause were approved for the purposes of scientific research by the appropriate board of the veterinary inspection office, Munich, Germany (Permit Number 8.175.10024.1319.3). Horses were treated according to the ethical principles and guidelines for scientific experiments on animals according to the Assosiation for Research in Vision and Ophthalmology (ARVO) statement for the use of animals in ophthalmic and vision research. No experimental animals were used in this study. Sampling of the human donor eyes was approved by the local ethics committee in compliance with the tenets of the declaration of Helsinki. The donor gave informed consent.

### 4.2. Preparation for Immunohistochemistry

Posterior eyecups of horses were immersion-fixed with Bouin’s solution (Sigma Aldrich, Deisenhofen, Germany). The human eye was obtained already fixed in formalin. The fixation was followed by dehydration in ascending alcohol series and slicing into pre-assigned fragments, as described before [29]. Afterwards, tissue blocks were embedded in paraffin, sectioned with a thickness of 8 µm and collected on coated slides (Superfrost Plus, Thermo Fisher Scientific, Bonn, Germany).

### 4.3. Immunohistochemistry of Target Tissue

Deparaffination of tissue sections was performed with xylol. Then, sections were rehydrated in descending alcohol series. Heat antigen retrieval was performed in citrate buffer at 99 °C for 15 min. To prevent unspecific antibody binding, sections were blocked with 1% bovine serum albumin in Tris-buffered saline-tween (TBS-T; 10 mM Tris, 150 mM NaCl, pH 7.2, 0.1% Tween 20) containing 5% normal goat serum for 40 min at room temperature. The next step was to incubate retina and RPE sections with primary antibody against peripherin 2 (mouse monoclonal antibody 5H2, directed against the 35-amino acid *C*-terminal segment of bovine peripherin 2, kindly provided by Robert S. Molday, Department of Biochemistry and Molecular Biology, University of British Columbia, Vancouver, BC, Canada; dilution 1:50) [26] and equine retina and RPE sections additionally with primary antibody against rhodopsin (rat monoclonal antibody supernatant 5G6, already characterized as cross-reactive with horse rhodopsin [30]) at 4 °C overnight, followed by a washing step with TBS-T. Then, sections were incubated with anti-mouse IgG Alexa Fluor 647 and anti-rat IgG Alexa Fluor 546 (both Invitrogen, Karlsruhe, Germany; dilution: 1:500), respectively, for 30 min at room temperature. Cell nuclei were counter-stained with 4',6-diamidino-2-phenylindole (DAPI; Invitrogen; dilution: 1:1000). Finally, sections were mounted with glass cover slips using Roti Mount Fluor Care (Carl Roth, Karlsruhe, Germany). Fluorescent images were recorded using Axio Imager M1 and Axio Vision 4.8 software (both Zeiss, Göttingen, Germany).

### 4.4. Separation of Equine RPE Cells for Immunocytochemistry

Eyes were prepared immediately after collection as described before [5]. Briefly, the first step was to remove the residual periocular tissue. Eye globes were cut open circumferentially, and anterior parts of the eye, vitreous and neurosensory retina were carefully removed. The RPE cell layer was then enzymatically detached from the underlying Bruch’s membrane and choroid by filling the eyecups with pre-warmed dissociation buffer (40 U/mL papain, Carl Roth; 1 mM PBS/EDTA pH 7.4; 260 mM l-cysteine, Sigma Aldrich; 1% bovine serum albumin) and incubated at 37 °C and 5% CO_2_ atmosphere for 25 min. Afterwards, RPE cells were resuspended in dissociation buffer inside the eyecup and transferred into DMEM (PAN-Biotech, Aidenbach, Germany) supplemented with 10% heat-inactivated fetal bovine serum (FCS) and 1% penicillin/streptomycin (P/S) (both PAN-Biotech) to block the enzymatic activity of papain. The next step was to centrifuge the suspension at 130× *g* for 5 min and wash the RPE cells two times. Finally, RPE cells were resuspended in DMEM containing 10% FCS and 1% P/S, seeded into T25 cell culture flasks (Sarstedt, Nümbrecht, Germany) and cultivated at 37 °C and 5% CO_2_ atmosphere until the cells were 90%–100% confluent. Subsequently, for cell passaging, medium was removed and cells were washed with PBS. To detach cells from the bottom of the flask, cells were incubated with Trypsin (0.05%)/EDTA (0.02%) (PAN-Biotech) at 37 °C for 5 min. Cells were then resuspended and collected in PBS and centrifuged at 130× *g* for 5 min to stop the reaction. Then, an appropriate number of cells was resuspended in medium, divided and transferred into a T75 cell culture flask. 

### 4.5. Preparation for Immunocytochemistry

Equine RPE cells were harvested with Trypsin (0.05%)/EDTA (0.02%) for 5 min, washed twice with PBS and centrifuged between washing steps at 4 °C, 130× *g* for 5 min. Then, they were seeded onto glass slides and allowed to attach and grow at 37 °C and 5% CO_2_ atmosphere for 48 h. Slides were rinsed in PBS, and after drying, they were fixed in ice cold acetone for 10 min. To prevent unspecific antibody binding, slides were blocked with 1% BSA in TBS-T and 5% normal goat serum for 40 min at room temperature. Fluorescence labelling and image acquisition was performed similarly as for immunohistochemistry of retina and RPE sections.

### 4.6. Quantification of Fluorescence Intensities

The fluorescence intensities of photoreceptor segments of 6 healthy and 6 diseased retina sections and the fluorescence intensities of RPE cells of 7 healthy and 8 diseased RPE sections were quantified using open source ImageJ 1.47 software (avaliabel online: http://imagej.nih.gov/ij/index.html). First, the area and the integrated density of the region of interest (Region 1) were measured. For background subtraction, the mean grey value of a region without fluorescence (Region 2) was measured. The following formula was used to calculate the corrected total fluorescence (CTF):

CTF = integrated density of Region 1 − (area of Region 1 × mean grey value of Region 2)
(1)

The mean value from healthy CTFs was formed and set to 100%. Statistical analyses were performed using the Kolmogorov–Smirnov test first to determine the data distribution. Since the fluorescence intensities of peripherin 2 of diseased RPE samples and controls were not normally distributed (Kolmogorov–Smirnov: *p* ≤ 0.05), the Mann–Whitney test was applied for the calculation of abundances. The data of the statistical analysis were considered as significant with a *p*-value of ≤0.05 (* *p* ≤ 0.05, ** *p* ≤ 0.01, *** *p* ≤ 0.001).

## 5. Conclusions

Investigations of the changes at the outer blood-retinal barrier in the case of uveitis at the molecular level enabled us to gain novel important insights into the sight-threatening disease, ERU. We demonstrated, for the first time, that peripherin 2 is obviously detectable in healthy human, as well as in healthy equine RPE. Peripherin 2 gets into RPE cells by phagocytosis similarly to rhodopsin, but is additionally expressed by healthy RPE cells themselves. Furthermore, expression changes of peripherin 2 selectively concern RPE cells, but not photoreceptor outer segments in uveitic cases, pointing to a disease-associated role of selective peripherin 2 downregulation in the outer blood retinal barrier. Novel localization and characterization of peripherin 2 in healthy and uveitic RPE cells is very interesting and sets the foundation for further examinations of the physiological and pathophysiological processes taking place in the RPE.
